# Clinical and microbiological efficacy of continuous versus intermittent application of meropenem in critically ill patients: a randomized open-label controlled trial

**DOI:** 10.1186/cc11405

**Published:** 2012-06-28

**Authors:** Ivan Chytra, Martin Stepan, Jan Benes, Petr Pelnar, Alexandra Zidkova, Tamara Bergerova, Richard Pradl, Eduard Kasal

**Affiliations:** 1 Department of Anesthesiology and Intensive Care Medicine, Charles University in Prague, Faculty of Medicine in Plzen, University Hospital Plzen, alej Svobody 80, 304 60, Czech Republic; 2 Department of Microbiology, Charles University in Prague, Faculty of Medicine in Plzen, University Hospital Plzen, alej Svobody 80, 304 60, Czech Republic

## Abstract

**Introduction:**

Meropenem bactericidal activity depends on the time when the free drug concentrations remain above the minimum inhibitory concentration of pathogens. The goal of this study was to compare clinical and bacteriological efficacy of continuous meropenem infusion versus bolus administration in critically ill patients with severe infection, and to evaluate the safety of both dosing regimens.

**Methods:**

Patients admitted to the interdisciplinary Intensive Care Unit (ICU) who suffered from severe infections and received meropenem were randomized either in the Infusion group (*n *= 120) or in the Bolus group (*n *= 120). Patients in the Infusion group received a loading dose of 2 g of meropenem followed by a continuous infusion of 4 g of meropenem over 24 hours. Patients in the Bolus group were given 2 g of meropenem over 30 minutes every 8 hours. Clinical and microbiological outcome, safety, meropenem-related length of ICU and hospital stay, meropenem-related length of mechanical ventilation, duration of meropenem treatment, total dose of meropenem, and ICU and in-hospital mortality were assessed.

**Results:**

Clinical cure at the end of meropenem therapy was comparable between both groups (83.0% patients in the Infusion vs. 75.0% patients in the Bolus group; *P *= 0.180). Microbiological success rate was higher in the Infusion group as opposed to the Bolus group (90.6% vs. 78.4%; *P *= 0.020). Multivariate logistic regression identified continuous administration of meropenem as an independent predictor of microbiological success (OR = 2.977; 95% CI = 1.050 to 8.443; *P *= 0.040). Meropenem-related ICU stay was shorter in the Infusion group compared to the Bolus group (10 (7 to 14) days vs. 12 (7 to 19) days; *P *= 0.044) as well as shorter duration of meropenem therapy (7 (6 to 8) days vs. 8 (7 to 10) days; *P *= 0.035) and lower total dose of meropenem (24 (21 to 32) grams vs. 48 (42 to 60) grams; *P *< 0.0001). No severe adverse events related to meropenem administration in either group were observed.

**Conclusions:**

Continuous infusion of meropenem is safe and, in comparison with higher intermittent dosage, provides equal clinical outcome, generates superior bacteriological efficacy and offers encouraging alternative of antimicrobial therapy in critically ill patients.

## Introduction

Severe infections in critically ill patients and increasing antibiotic resistance are major healthcare problems affecting morbidity and mortality in the intensive care unit. Antibacterial drug discovery and development have slowed considerably in recent years. The number of new antibacterial medicines entering the clinic has been declining and, in view of this fact, new compounds for multi-drug resistant Gram-negative bacilli will unlikely be available for more than 10 years [1,2]. The problems associated with escalating resistance and decreased development of antibiotics with novel mechanisms of action has required more research into existing antibiotics. The effort to maximize antibiotic activity has led in recent years to the interest for optimal dosing based on pharmacodynamic and pharmacokinetic properties of antibiotics [3].

Because of broad spectrum activity against Gram-negative and -positive organisms and low toxicity, meropenem remains a suitable choice for treatment of severe infections in critically ill patients. It is currently established that meropenem, like other β-lactam antibiotics, displays time-dependent bactericidal activity and the percentage of the dosing interval that free drug concentrations remain above the minimum inhibitory concentration of pathogen (%fT > MIC) is the most important parameter for predicting their antibacterial efficacy [4,5]. As a minimum standard for carbapenems the T > MIC should be maintained at 40%, [6]. However, patients with serious bacterial infections with T > MIC of 100% displayed significantly greater clinical cure (82% vs. 33%; *P *= 0.002) and bacteriological eradication (97% vs. 44%; *P *< 0.001) than patients with T > MIC of < 100%, thus maintaining antibiotic concentrations above the MIC for 100% of the dosing interval should be considered [7,8]. The maximum killing effect of β-lactams is reached at four to five times the MIC with higher concentration not contributing further to increasing the antimicrobial effect. It can be presumed that intermittent infusion recommended by pharmaceutical companies results in high peak concentration and low through concentration and can cause reduced efficacy. Furthermore, pathophysiological changes that occur in seriously ill patients with sepsis often affect volume of distribution, drug clearance and altered pharmacokinetic parameters making these recommendations potentially inappropriate [9-12]. The use of continuous administration of β-lactams was studied in some trials [13-22], but strong evidence of clinical efficacy of this alternative is lacking.

The goal of this study was to compare the clinical and bacteriological efficacy of continuous infusion of meropenem versus traditional bolus administration in critically ill patients with severe infection, and to evaluate the safety of both dosing regimens.

## Materials and methods

This was a single-center, prospective, randomized, open-label comparative study conducted at the interdisciplinary ICU of Department of Anesthesiology and Intensive Care Medicine at Charles University teaching hospital in Plzen. The trial was approved by the local research ethics committee (Ethics Committee of University Hospital in Plzen). Informed consent was not required because the protocol of meropenem administration was considered the standard of the routine clinical practice in this intensive care unit (ICU) and no interventions were made during data collection and analysis. Nevertheless, subjects were informed at discharge that they had participated in this clinical study and subsequent written consent was obtained.

All patients admitted to the 11-bed interdisciplinary ICU between September 2007 and May 2010 who suffered, at admission or during the ICU stay, from severe infection and received meropenem with predicted duration of treatment for at least four days were considered for inclusion. All enrolled patients fulfilled the criteria of sepsis [23]. Types of infections included abdominal, respiratory, skin and soft tissue, bloodstream, central nervous system (CNS), urinary tract or other sources of infections. Diagnosis of infection was established in accordance with predefined criteria [24]. Meropenem administration was indicated mostly as second-line antimicrobial therapy based on microbiological findings but also as empirical therapy for sepsis without a proven source and pathogen. Concomitant antimicrobial therapy was allowed. The absolute majority of antimicrobial therapy (meropenem and concomitant) was started according to consultation and stewardship of a university hospital microbiologist. The MIC of meropenem in identified pathogens was determined by using E test (AB BIODISK, Solna, Sweden) methodology.

Exclusion criteria included age younger than 18 years, pregnancy, acute or chronic renal failure with glomerular filtration rate lower than 0.5 ml/s, immunodeficiency or immunosuppressant medication, neutropenia (absolute neutrophil count < 1,000 cells/mm^3^), and hypersensitivity or allergy to meropenem. Glomerular filtration rate (GFR) for all patients was daily calculated by using of the four-variable Modification of Diet in Renal Disease (MDRD) formula [25]. For patients who developed renal impairment (GFR < 0.5 ml/s) during the study, the dosage of meropenem was adjusted according to product labeling [26]. Antibiotic therapy was stopped at improvement of the clinical state and signs of subsidence of infection (body temperature below 38, 3°C for more than 24 hours, white blood cells (WBC) count less than 11,000/mm^3 ^or decrease by 25% of maximal value) [27,28].

### Patient's randomization, protocol and outcome measures

Patients meeting inclusion criteria with no presence of exclusion criteria were randomized using sealed opaque envelopes in one-to-one proportion without stratification to receive either continuous infusion (Infusion group, *n *= 120) or intermittent intravenous application (Bolus group, *n *= 120) of open-label meropenem (Meronem; AstraZeneca Pharmaceuticals, Macclesfield, Cheshire, UK). The patients in the Infusion group received a loading dose of 2 g of meropenem in 20 ml of normal saline infused by central line over 30 minutes followed immediately by continuous infusion of 4 g of meropenem over 24 hours. With the respect to stability of meropenem, the infusion was given as four 1 g infusions over six hours in 50 ml of normal saline [29,30]. The patients in the Bolus group received 2 g of meropenem in 20 ml of normal saline infused by central line over 30 minutes every 8 hours. In both groups, meropenem was administered through separate lumen of central venous catheter using a perfusor (Perfusor^® ^fm (MFC); B. Braun, Melsungen AG, Germany). Patients in both groups were treated during their ICU stay by the regular team of ICU physicians and received standard intensive care.

Primary outcome measures were clinical and microbiological efficacy of meropenem therapy. In *post-hoc *subanalyses, clinical and microbiological outcomes were evaluated in subgroups of patients with empiric and culture-based therapy, in patients without concomitant therapy potentially active against Gram-negative bacteria (GNB), in patients with APACHE II > 20 and in patients with MICs of pathogens higher or equal 1.5 mg/l. The clinically evaluable population included patients who met the protocol definition, received meropenem therapy for at least four full days and had sufficient data available to determine clinical outcome at the end of treatment (EOT). Microbiologically evaluable patients had at least one bacterial pathogen identified at baseline that was susceptible or intermediate to meropenem.

Clinical response was evaluated at the end of therapy as treatment success or failure. Clinical success was defined as complete or partial resolution of leukocytosis, temperature, and clinical signs and symptoms of infection (defined as cure or improvement). Failure consisted of any of the following: persistence or progression of signs and symptoms of infection, development of new clinical findings consistent with active infection, or death from infection. The addition of an agent for Gram-positive bacteria coverage was not considered a clinical failure. When a situation was unclear, two investigators independently determined the status. If their initial assessments differed, the third independent physician arbitrated the result.

A microbiologist blinded to the patient allocation assessed microbiological outcomes at the end of the meropenem therapy. The categories of eradication and presumed eradication were deemed as microbiological success. Persistence, presumed persistence and resistance were designated as microbiologic failure. For patients with multiple organisms at the same infection site, microbiologic evaluation was based on organisms susceptible to meropenem. Detection of a new pathogen from the site of infection during the meropenem therapy was evaluated as colonization if no new antibiotic therapy was indicated, or as superinfection if antibiotic therapy to target the results of the new culture was established. Patients who developed colonization were classified as microbiological successes; patients with superinfection caused by pathogens involved in therapeutic spectrum of meropenem were assessed as microbiological failures. Superinfections caused by *methicillin-resistant staphylococci *(MRSA), *Enterococcus faecium, Candida *spp. and other fungal species were not classified as microbiological failure. The decision between colonization and superinfection was accomplished according to the consultation between a microbiologist and treating clinician. The outcome categories exact definitions used are described in the additional file (Additional file 1, Table S1).

Secondary outcome measures were meropenem-related length of mechanical ventilation, meropenem-related length of ICU and hospital stay (LOS), ICU and in-hospital mortality, duration of meropenem treatment, the total dose of meropenem and the safety of both dosing regimens. Meropenem-related length of mechanical ventilation was defined as the number of days of mechanical ventilation from the start of meropenem administration. Meropenem-related length of stay was defined as the number of days from the beginning of meropenem therapy to the day of discharge from ICU or from the hospital. Safety of meropenem therapy was evaluated by clinical symptoms (diarrhea, rash, vomiting and seizures) during the meropenem therapy and by assessment of laboratory parameters and their changes during meropenem therapy (transaminases, alkaline phosphatase, bilirubin, thrombocytes).

Other data, such as age, weight, gender, APACHE II (Acute Physiology and Chronic Health Evaluation II) and SOFA (Sequential Organ Failure Assessment) scores at the start of meropenem therapy, diagnostic group, type and etiology of infection treated by meropenem, MICs of identified pathogens, length of ICU stay prior to meropenem administration, occurrence and length of previous antimicrobial therapy, number of antibiotics given before the start of meropenem, the rate of empiric and culture-based meropenem therapy and the rate of community and nosocomial infection were recorded. C-reactive protein (CRP), white blood cell (WBC) count, platelet count, aspartate aminotransferase (AST), alanine aminotransferase (ALT), alkaline phosphatase (ALP) and total bilirubin were examined at the start and at the end of meropenem therapy.

### Statistical analysis

Baseline demographic characteristics, safety and drug-related adverse events, including laboratory results, were assessed on an intention-to-treat (ITT) basis. The secondary clinical outcome measures were analyzed on both, ITT and per protocol basis. The clinical cure rate, concomitant antibiotic therapy, microbiological findings and bacteriological success rate were evaluated only in the per protocol population.

Sample size calculations were based on clinical success rates in similar previous studies with continuous application of meropenem and other β-lactam antibiotics presenting clinical and microbiological outcome improvement by 12% to 39% in the population [17,31,32]. We calculated a study size of 95 patients in each group by postulating an improvement of clinical outcome in a continuous group by 20% for two-sided tests with type I error of 5% and power of 90%. Because of a predictable loss of 15% to 20% of patients entering the study due to violation of protocol, we proposed to increase the number of patients in each group to 120. The Kolmogorov-Smirnov test was used to check for normal distribution of data. Continuous normally distributed data were tested with paired or unpaired t test, not normally distributed data using Mann-Whitney U test and Wilcoxon signed-rank test. Categorical data were tested using chi-square and Fisher's exact test. Multiple logistic regression with backward stepwise variable selection was used to identify the variables independently associated with the clinical and microbiological outcome of meropenem therapy in the clinically and microbiologically evaluable patients. Factors with *P *< 0.20 according to univariate analysis were entered into the multivariate model. To evaluate model calibration, we performed the Lemeshow-Hosmer goodness-of-fit test. The predictive accuracy of the multivariate model was expressed as the area under the receiver operating characteristic (ROC) curve. Results of logistic regression are reported as odds ratios (OR) with 95% confidence intervals (CI). Unless stated otherwise, normally distributed data are presented as mean ± standard deviation, and as median (interquartile ranges) where not normally distributed. Relative risk (RR) is presented with 95% CI. A *P *< 0.05 was considered statistically significant for all tests. All calculations were performed with MedCalc^® ^version 11.6.1.0. (Frank Schoonjans, MedCalc Software, Broekstraat 52, 9030 Mariakerke, Belgium).

## Results

**Figure 1:**
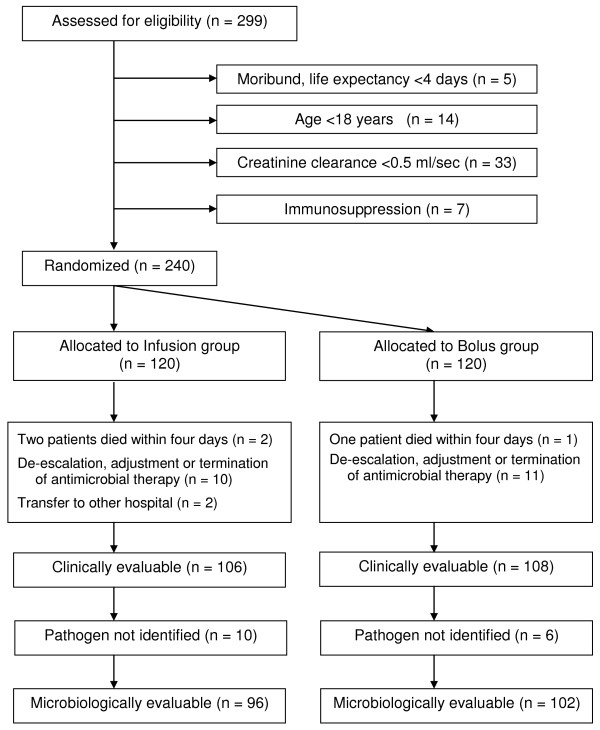
**Flow of participants through the trial**.

A total of 299 patients were screened for eligibility during the study period (Figure 1). Fifty-nine patients met any of the exclusion criteria, 120 patients were randomized to the Infusion group and 120 patients to the Bolus group. Fourteen patients of the Infusion group and 12 patients of the Bolus group were excluded from the per protocol evaluation because of death or other violation of protocol. Reasons for drop out in both groups were similar. One hundred and six patients in the Infusion group and 108 patients in the Bolus group were found clinically evaluable. Because of the failure to identify causative pathogen (negative cultures from the presumed site of infection) in 10 patients from the Infusion and in 6 patients from the Bolus group, only 96 patients in the Infusion group and 102 patients in the Bolus group were selected for microbiological evaluation (Figure 1).

Using ITT analysis both groups were well matched for demographics and baseline characteristics. No significant differences in severity of illness (APACHE II and SOFA scores), in the type of infection and other evaluated parameters were observed (Table 1), except for the trend to a lower number of surgical patients (*n *= 18, (15.0%) vs. *n *= 30, (25.0%); *P *= 0.075), and longer length of ICU stay before the start of meropenem therapy in the Infusion group (9.0 (5.0 to 16.0) days vs. 7.0 (3.0 to 11.0) days; *P *= 0.036).

**Table 1:** Demographics and baseline characteristics of randomized patients

Characteristics	Infusion group (*n *= 120)	Bolus group (*n *= 120)	*P-*value
Age (years)	44.9 ± 17.8	47.2 ± 16.3	0.503
Male	78 (65.0%)	83 (69.2%)	0.583
Weight (kg)	78 (70.0 to 90.0)	80 (72.0 to 95.5)	0.079
APACHE II	21.4 ± 7.9	22.1 ± 8.79	0.545
SOFA	10.4 ± 2.9	10.6 ± 3.5	0.738
GFR calculated by MDRD (ml/s)	1.20 (0.77 to 1.80)	1.19 (0.88 to 1.59)	0.813
Diagnostic group n (%)			
Cardiac surgery	4 (3.3%)	2 (1.7%)	0.685
Neurology and neurosurgery	3 (2.5%)	3 (2.5%)	1,000
Surgery	18 (15.0%)	30 (25.0%)	0.075
Gastroenterology	22 (18.3%)	14 (11.7%)	0.205
Traumatology	43 (35.8%)	45 (37.5%)	0.788
Respiratory failure	2 (1.7%)	7 (5.8%)	0.171
Circulatory failure	24 (20.0%)	18 (15.0%)	0.396
Other	4 (3.3%)	1 (0.8%)	0.370
Type of infection n (%)			
Not identified	0 (0.0%)	2 (1.6%)	0.498
Abdominal	23 (19.2%)	31 (25.8%)	0.357
Respiratory	66 (55.0%)	61 (50.8%)	0.605
Soft tissues, skin	5 (4.2%)	6 (5.0%)	1.000
Bloodstream	10 (8.3%)	11 (9.2%)	1.000
Uroinfection	11 (9.2%)	6 (5.0%)	0.314
Central nervous system	3 (2.5%)	2 (1.6%)	1.000
More sources	2 (1.6%)	1 (0.8%)	1.000
Nosocomial infection	110 (91.7%)	113 (94.2%)	0,616
Concomitant ATB therapy n (%)	58 (48.3%)	61 (50.8%)	0.796
Concomitant ATB therapy potentially active against GNB	11 (9.2%)	6 (5.0%)	0.314
Previous ATB therapy n (%)	114 (95.0%)	113 (94.2%)	1.000
Previous ATB therapy (days)	7.0 (6.0 to 8.0)	7.0 (6.0 to 8.0)	0.338
Number of previous ATB	3 (3 to 5)	4 (3 to 5)	0.509
Empiric therapy n (%)	6 (5.0%)	7 (5.8%)	1.000
Length of ICU stay before meropenem therapy (days)	9.0 (5.0 to 16.0)	7.0 (3.0 to 11.0)	**0.036**
Leucocytes (x 10^9^/L)	15.9 (11.5 to 21.1)	14.1 (11.2 to 19.6)	0.437
CRP (mg/L)	155 (115 to 197)	199 (117 to 262)	0.090

Values are presented as absolute (percentage) or mean ± standard deviation or median (interquartile range). APACHE II, Acute Physiology and Chronic Health Evaluation II; ATB, antibiotic; CRP, C-reactive protein; GFR, Glomerular filtration rate; GNB, Gram-negative bacteria; ICU, intensive care unit; MDRD, Modification of Diet in Renal Disease; SOFA, Sequential Organ Failure Assessment.

The type, the rate and MICs of isolated pathogens in clinically evaluable patients were similar (Table 2). The dominant isolated pathogen in both groups was *Klebsiella *spp. (59.4% vs. 46.3%; *P *= 0.057).

**Table 2:** Bacterial isolates and susceptibility characteristics to meropenem in clinically evaluable population

	Number n (%)			MIC (mg/l)		
				
Pathogen^1^	Infusion group *n *= 106	Bolus group *n *= 108	*P*	Infusion group *n *= 106	Bolus group *n *= 108	*P*
None cultured	10 (9.4%)	6 (5.6%)	0.310	n/a	n/a	n/a
*Acinetobacter *spp.	8 (7.5%)	12 (11.1%)	0.482	2.000 (1.000 to 2.000)	1.500 (1.500 to 2.000)	0.635
*Citrobacter *spp.	3 (2.8%)	4 (3.7%)	1.000	0.190 (0.092 to 0.422)	0.125 (0.040 to 0.470)	0.724
*Enterobacter *spp.	3 (2.8%)	7 (6.5%)	0.332	0.190 (0.093 to 0.331)	0.060 (0.053 to 0.090)	0.170
*Escherichia coli*	6 (5.7%)	9 (8.3%)	0.594	0.090 (0.090 to 0.120)	0.120 (0.072 to 0.380)	0.260
*Klebsiella *spp.	63 (59.4%)	50 (46.3%)	0.057	0.120 (0.090 to 0.130)	0.090 (0.060 to 0.130)	0.205
*Morganella morganii*	3 (2.8%)	5 (4.6%)	0.721	0.250 (0.250 to 0.542)	0.320 (0.250 to 0.535)	0.760
*Pseudomonas aeruginosa*	4 (3.8%)	6 (5.6%)	0.748	2.000 (1.750 to 2.000)	1.750 (1.000 to 2.000)	0.363
*Serratia marcescens*	4 (3.8%)	6 (5.6%)	0.748	0.110 (0.094 to 0.222)	0.125 (0.094 to 0.190)	0.830
*Stenotrophomonas maltophilia*	2 (1.9%)	3 (2.8%)	1,000	2.000 (2.000 to 2.000)	2.000 (1.625 to 2.000)	0.519
MIC of all microorganisms				0.125 (0.090 to 0.640)	0.130 (0.090 to 0,350)	0.970

Values are presented as absolute (percentage) or median (interquartile range).

^1 ^Isolates do not include all pathogens, but those treated with meropenem; MIC, minimum inhibitory concentration; n/a, not applicable

Concomitant antibiotic therapy was used in more than 50% of clinically evaluable patients in the Infusion and Bolus groups. There were no differences in the rate of combined antimicrobial therapy and in the type of concomitant antibiotics (Table 3). The group of penicillins involved oxacillin, ampicillin and piperacillin/tazobactam, aminoglycosides comprised gentamicin and amikacin, fluorochinolons included ciprofloxacin and levofloxacin and the antifungal drugs were represented by fluconazol, voriconazole and amphotericin b. The concomitant antibiotic therapy potentially active against GNB was given in 11 patients in the Infusion group (3 - piperacillin/tazobactam + gentamicin, 1 - piperacillin/tazobactam, 1 - amikacin, 5 - ciprofloxacin, 1- levofloxacin) and in 6 patients in the bolus group (2-piperacillin/tazobactam, 1 - gentamicin and 3 - ciprofloxacin; *P *= 0.314).

**Table 3:** Concomitant antimicrobial therapy in clinically evaluable population

Parameter	Infusion group (*n *= 106)	Bolus group (*n *= 108)	*P-*value
Any	51 (48.1%)	55 (50.9%)	0.685
Penicillins	11 (10.4%)	5 (4.6%)	0.126
penicillin G	3	1	0.367
oxacillin	4	2	0.443
piperacillin/tazobactam	4	2	0.443
Aminoglycosides	4 (3.8%)	1 (0.9%)	0.210
Fluoroquinolones	6 (5.7%)	3 (2.8%)	0.330
Vancomycin	4 (3.8%)	11 (10.2%)	0.106
Linezolid	11 (10.4%)	7 (6.5%)	0.335
Metronidazole	7 (6.6%)	13 (12.0%)	0.240
Antifungals	34 (32.1%)	30 (27.6%)	0.551

Values are presented as absolute (percentage).

Linezolid or vancomycin for MRSA infection was indicated in the Infusion group in seven patients (two abdominal, three respiratory and two blood stream infection) and in five patients (two abdominal, one respiratory and two blood stream infection) in the Bolus group (*P *= 0,768). Rate and indication of antimycotic therapy in both groups was similar. Etiology of *Candida s*pp. infection in the Infusion group was proven in 33 patients (16 abdominal, 13 respiratory and 4 urine infections) and in 30 patients (17 abdominal, 11 respiratory, 1 bloodstream and 1 urine infection) in the Bolus group (*P *= 0.654). *Aspergillus *spp. etiology of respiratory infection was confirmed in one patient in the Infusion group.

The rate of clinical cure and improvement in clinically evaluable patients at EOT (Table 4) was comparable between the treatment groups (88 patients (83.0%) in the Infusion group vs. 81 (75.0%) patients in the Bolus group; *P *= 0.180; RR = 1.1069; 95% CI = 0.9634 to 1.2718). We did not observe any difference in other evaluated subgroups of patients except for patients with culture-based therapy, in which we found the trend for better clinical outcome in the Infusion group (86.0%) against the Bolus group (74.3%); (*P *= 0,051; RR = 1.1581; 95% CI = 1.007 to 1.3314). In patients with intraabdominal infections a trend towards higher clinical success rate was observed in the Infusion group (15 patients (83.3%) vs. 15 (55.6%); *P *= 0.063; RR = 1.500; 95% CI = 1.010 to 2.228) (Table 4). Shorter meropenem-related ICU stay was detected in both ITT and in the clinically evaluable population in the Infusion compared to the Bolus group (10 (7 to 14) days vs. 12 (7 to 19) days; *P *= 0.044) and (10 (7 to 16.5) days vs. 13 (8 to 21) days; *P *= 0.042), respectively (Table 5), but no difference was found in subgroups of patients with APACHE II > 20 and patients with MICs of pathogen ≥ 1.5 mg/l (Additional file 2, Table S2). The shorter duration of meropenem therapy (7 (6 to 8) days vs. 8 (7 to 10) days; *P *= 0.035) and lower total dose of meropenem (24 (24 to 32) grams vs. 48 (42 to 60) grams; *P *< 0.0001) were observed in the Infusion group (Table 5). The CRP concentration at the end of meropenem therapy evaluated in *post-hoc *analysis was higher in the Bolus group in overall clinically evaluable patients (59 (32 to 87) mg/l vs. 72 (37 to 114) mg/l; *P *= 0.037) and in the subgroup of patients with APACHE II > 20 (62 (32 to 87) mg/l vs. 78 (42 to 126) mg/l; *P *= 0,036) (Additional file 2, Table S2).

**Table 4:** Clinical cure rates in clinically evaluable patients at the end of treatment.

	Infusion, n (%)	Bolus, n (%)	*P-*value
**Overall**	88/106 (83.0%)	81/108 (75.0%)	0.180
Cured	30/106 (28.3%)	24/108 (21.3%)	0.347
Improved	58/106 (54.7%)	57/108 (52.8%)	0.786
Culture-based therapy	86/100 (86.0%)	75/101 (74.3%)	**0.051**
Empiric therapy	4/6 (66.7%)	6/7 (85.7%)	0.560
Without concomitant ATB therapy potentially active against GNB	80/95 (84.2%)	76/102 (74.5%)	0.114
APACHE II > 20	37/49 (75.5%)	42/53 (79.2%)	0.813
MIC ≥ 1.5 mg/l	10/14 (71.4%)	12/21 (57.1%)	0.488
**Type of infection**			
Not identified	0	1/1 (100%)	1.000
Abdominal	15/18 (83.3%)	15/27 (55.6%)	0.063
Respiratory	52/58 (89.7%)	47/56 (83.9%)	0.416
Soft tissues, skin	4/5 (80.0%)	3/4 (75.0%)	1.000
Bloodstream	7/9 (77.8%)	8/11 (72.7%)	1.000
Urinary tract	7/11 (63.6%)	5/6 (83.3%)	0.600
CNS	3/3 (100%)	2/2 (100%)	n/a
More sources	2/2 (100%)	1/1 (100%)	n/a

Values are presented as absolute (percentage). APACHE II, Acute Physiology and Chronic Health Evaluation II; ATB, antibiotic; CNS, central nervous system; GNB, Gram-negative bacteria; MIC, minimum inhibitory concentration; n/a, not applicable

**Table 5:** Outcome in ITT and clinically evaluable patients

Parameter	Infusion	Bolus	*P-*value
Number of patients			
ITT	120	120	
Clinically evaluable	106	108	

Meropenem-related length of mechanical ventilation			
ITT	9 (5 to 13)	11 (6 to 17)	0.051
Clinically evaluable	9 (5 to 15)	12 (6 to 19)	0.058
Meropenem-related ICU LOS			
ITT	10 (7 to 14)	12 (7 to 19)	**0.044**
Clinically evaluable	10 (7 to 16.5)	13 (8 to 21)	**0.042**
Meropenem-related hospital LOS			
ITT	26 (17 to 38)	22 (12 to 35)	0.079
Clinically evaluable	28 (18 to 39)	25 (14 to 42)	0.412
ICU mortality			
ITT	18 (15.0%)	25 (20.8%)	0.313
Clinically evaluable	14 (11.6%)	17 (14.2%)	0.701
Hospital mortality			
ITT	21 (17.5%)	28 (23.3%)	0.337
Clinically evaluable	17 (16.0%)	19 (15.7%)	0.857
Duration of meropenem therapy (days)			
ITT	7 (5 to 8)	8 (6 to 9)	**0.041**
Clinically evaluable	7 (6 to 8)	8 (7 to 10)	**0.035**
Total dose of meropenem (g)			
ITT	24 (21 to 32)	48 (42 to 48)	**< 0.0001**
Clinically evaluable	24 (24 to 32)	48 (42 to 60)	**< 0.0001**

Values are presented as absolute (percentage) or median (interquartile range). ICU, intensive care unit; ITT, intention-to-treat; LOS, length of stay

Microbiological success rate (assessed in microbiologically evaluable patients only) was higher in the Infusion group (87 patients with overall pathogen eradication (90.6%) vs. 80 (78.4%); *P *= 0.020; RR = 1.156; 95% CI = 1.024 to 1.303). In the subanalyses we detected similar results in subgroups of patients with culture-based therapy and in patients without concomitant antibiotic therapy potentially active against GNB (Table 6.).

**Table 6:** Microbiological success rates in microbiologically evaluable patients

	Infusion group, n (%)	Bolus group, n (%)	*P-*value
**Eradication overall**	87/96 (90.6%)	80/102 (78.4%)	**0.020**
Verified eradication	67/96 (69.8%)	62/102 (60.8%)	0.233
Presumed eradication	20/96 (20.8%)	18/102 (17.6%)	0.592
Culture-based therapy	82/90 (91.1%)	74/95 (77.9%)	**0.015**
Empiric therapy	5/6 (83.0%)	5/7 (71.4%)	1.000
Without concomitant ATB therapy potentially active against GNB	77/85 (90.6%)	75 (78.1%)	**0.026**
APACHE II > 20	41/47 (87.2%)	36/49 (73.5%)	0.125
MIC ≥ 1.5 mg/l	11/14 (78.6%)	13/21 (61.9%)	0.292
*Acinetobacter *spp.	7/8 (87.5%)	9/12 (75%)	0.619
*Citrobacter *spp.	3/3 (100%)	3/4 (75%)	1.000
*Enterobacter *spp.	3/3 (100%)	7/7 (100%)	n/a
*Escherichia coli*	5/6 (83.3%)	7/9 (77.7%)	1.000
*Klebsiella *spp.	58/63 (92.1%)	39/50 (78.0%)	0.055
*Morganella morganii*	3/3 (100%)	5/5 (100%)	n/a
*Pseudomonas aeruginosa*	3/4 (75%)	3/6 (50%)	0.571
*Serratia marcescens*	4/4 (100%)	6/6 (100%)	n/a
*Stenotrophomonas maltophilia*	1/2 (50%)	1/3 (33.3%)	1.000
**Persistence overall**	9/96 (9.4%)	22/102 (21.6%)	**0.020**
*Acinetobacter spp*.	0/8 (0%)	2/12 (16.7%)	0,495
*Citrobacter *spp.	0/3 (0%)	1/4 (25.0%)	1.000
*Escherichia coli*	1/6 (16.7%)	2/9 (22.2%)	1.000
*Klebsiella *spp.	5/63 (7.9%)	11/50 (22.0%)	0.055
*Pseudomonas aeruginosa*	0/4 (0%)	2/6 (33.3%)	0.491
*Stenotrophomonas maltophilia*	1/2 (50.0%)	0/3 (0%)	0.400
**Resistance**	2/96 (2.1%)	4/102 (3.9%)	0.684
*Acinetobacter spp*.	1/8 (12.5%)	1/12 (8.3%)	1.000
*Pseudomonas aeruginosa*	1/4 (25.0%)	1/6 (16.7%)	1.000
*Stenotrophomonas maltophilia*	0/2 (0%)	2/6 (33.3%)	1.000

Values are presented as absolute (percentage). APACHE II, Acute Physiology and Chronic Health Evaluation II; ATB, antibiotic; GNB, Gram-negative bacteria;

MIC, minimum inhibitory concentration

In single pathogens, we observed a trend to better efficacy of infusion of meropenem in infections with *Klebsiella *spp. etiology (58 eradications (92.1%) vs. 39 (78.0%); *P *= 0.055; RR = 1.180; 95% CI = 1.002 to 1.391) (Table 6). Similar results in the rate of overall persistence and resistance were found with no differences in single pathogens (Table 6). The rate of colonization and superinfection during meropenem therapy did not differ (Additional file 3, Table S3). None of the isolates of meropenem therapeutic spectrum cultured from the site of infection during meropenem therapy required the initiation of new antibiotic therapy at EOT and all of these findings were classified as colonization. Superinfections with fungal species etiology necessitated antimycotic therapy but according to protocol were not evaluated as microbiological failure.

In multivariate logistic analysis, the number of antibiotics given before the start of meropenem (OR = 0.478; 95% CI = 0.323 to 0,706; *P *= 0.0002) and the age (OR = 0.947; 95% CI = 0.917 to 0.973; *P *= 0.0001) were negatively associated with clinical success of meropenem therapy. Continuous administration of meropenem was independently related to the microbiological success (OR = 2.977; 95% CI = 1.050 to 8.443; *P *= 0.040). Predictors of microbiological failure were the abdominal infection (OR = 0.249; 95% CI = 0.092 to 0,674; *P *= 0.006) and the number of antibiotics given before the start of meropenem (OR = 0.581; 95% CI = 0.383 to 0.881; *P *= 0.011). The performance of the final multivariate logistic models for clinical and microbiological outcome was good (Lemeshow-Hosmer goodness-of fit test for clinical and microbiological outcome with *P *= 0.63 and *P *= 0.53, respectively), and discriminated reasonably well between success and failure of meropenem therapy. The area under the ROC curve for clinical and microbiological outcome was 0.80, and 0.78, correctly predicting clinical and microbiological outcome (82.97% and 85.3%, respectively).

Possible meropenem-related clinical adverse events were rare and the rate in both groups did not differ (Additional file 4, Table S4). In laboratory parameters related to the safety of meropenem, we observed no difference between groups, although there was considerable increase in concentration of ALP at EOT in both groups against the baseline. The platelet count rose in the Bolus group significantly (Additional file 5, Table S5).

## Discussion

In our study, continuous infusion of four grams of meropenem per day preserved a similar rate of clinical cure, but reached a higher antimicrobial efficacy compared to intermittent bolus administration of six grams of meropenem divided in three doses. Multiple logistic regression identified the infusion of meropenem as an independent predictor of microbiological success. As far as we know, our study provides the largest patient-data pool concerning the issue of dosing regimens of meropenem and their microbiological and clinical efficacy in the critically ill. Kojika *et al. *[33], compared prolonged and bolus administration of meropenem in 10 patients with abdominal sepsis (5 in each group). Apart from early improvement of systemic inflammatory reactive syndrome (SIRS) score, no changes in body temperature, white blood cell count and serum CRP were demonstrated. In a retrospective cohort study in patients with ventilator associated pneumonia (VAP), Lorente *et al. *[17] observed significant clinical improvement in the infusion group (90.5% vs. 59.6%; *P *< 0.001). Intermittent application of 1 g of meropenem every six hours in this study might not reach required effective T > MIC and contribute to worse clinical outcome. Nicasio *et al. *[34] in a prospective, observational evaluation with a historical control group in patients with VAP presented reduced infection-related mortality (8.5% vs. 21.6%; *P *= 0.029), infection-related length of stay (11.7 ± 8.1 vs 26.1 ± 18.5; *P *= 0.001), and fewer events of superinfection (16% vs. 35.1%; *P *= 0.007) in patients treated by administration of three-hour infusions of cefepime 2 g every eight hours or meropenem 2 g every eight hours plus tobramycin and vancomycin. The retrospective and/or historical control design of these encouraging studies strongly affects their power.

Alas, our study failed to prove the primary outcome hypothesis of better clinical cure in the Infusion group (83% vs. 75%; *P *= 0.180). We have observed only a trend towards better clinical outcome in patients with abdominal infection (83.3% vs. 55.6%; *P *= 0.063). Some factors could contribute to these negative results. First, our sample size calculation was based on previously published results (Grant 2002, Lorente 2007), but these seem to be overly optimistic. Second, more than half of evaluated groups comprised post-surgery or trauma patients and other factors could play a role in their outcome. Nevertheless, the major cause of the failure to prove better clinical outcome in the Infusion group consists probably in the disproportion of daily cumulative dose of meropenem between groups (four grams in the Infusion and six grams in the Bolus group) and little difference between both types of meropenem administration in achieving T > MIC in relatively susceptible pathogens. We calculated the dose of meropenem (2 g every eight hours) in the Bolus group according to the suggestion for achievement antibiotic's optimal PTA (probability of target attainment) and CFR (cumulative fraction of response) in critically ill patients with serious infections [10,35-39], but the MICs in greater part of isolated pathogens (84.3%) were very low, which prolonged the effective time in the intermittent dosing regimen. Only in 15.7% of pathogens (*Acinetobacter *spp., *Pseudomonas aeruginosa *and *Stenotrophomonas maltophilia*) were MICs higher (2.000 (1.500 to 2.000) mg/l) (Table 2). Although there was no difference in overall clinical outcome, some laboratory markers of infection stress could indicate better efficacy of continuous treatment. Despite similar values of WBC and CRP at the start of therapy, the CRP levels at EOT were lower in the Infusion group (*P *= 0.037) (Additional file 2, Table S2).

Notwithstanding the same clinical outcome, we observed the trend towards shorter meropenem-related length of mechanical ventilation and significant shorter meropenem-related length of ICU stay in the Infusion group (Table 5). Similar results in comparable β-lactams studies demonstrated Nicasio *et al. *2010 (shorter infection-related stay), Lodise *et al. *2007 and Merchant *et al. *2008 (both shorter hospital stay) [34,40,41]. We did not find a difference in hospital LOS; however, the results in both groups were confounded by prolonged hospitalization of several patients with severe critical illness polyneuromyopathy and other complications in consequence of severe multiple organ failure. Hospital and ICU mortality in the Infusion group in ITT and in the clinically evaluable population was somewhat lower but not significantly, contrary to results of some nonrandomized studies [34,40,42].

Continuous administration of meropenem was associated with improvement of bacteriological efficacy documented as higher eradication rate and lower bacterial persistence (Table 6). A possible explanation for better microbial clearance in the infusion group could be the maldistribution of blood flow in the microcirculation in critically ill patients. Continuous application of meropenem can probably maintain higher concentrations of meropenem in the tissues than intermittent bolus dosing [43].

To date, no study comparing microbiological outcome of both dosing regimens of meropenem has been published. Two studies showed an improved bacteriological efficacy of continuous application of beta-lactams (ceftriaxone [44] and piperacillin/tazobactam [31], but also conflicting results were published [45-47].

No difference between both groups was observed in the rate of resistance, colonization and superinfection in our study (Additional file 3, Table S3). The occurrence of resistance in infusion and bolus group was low (2.1% vs. 3.9%) and developed in typical pathogens with lower susceptibility to meropenem (*Acinetobacter *spp., *Pseudomonas aeruginosa*, *Stenotrophomonas maltophilia*) (Table 6).

The representation of antibiotics given as concomitant therapy in both groups was similar. The high rate of concomitant antifungal drugs (32.1% in the Infusion and 27.6% in the Bolus group) was undoubtedly associated with previous antibiotic therapy (95% in the Infusion and 94.2% in the Bolus group) and related to preceding ICU stay prior to meropenem therapy was commenced.

The most commonly reported drug-related adverse events in patients treated with meropenem include diarrhea, rash, seizures and/or nausea and vomiting [29,48]. We did not evaluate the nausea, because all randomized patients in our study were sedated and mechanically ventilated. Meropenem administration was generally well tolerated and the incidence and types of adverse events possibly related to meropenem were similar in both treatment groups (Additional file 4, Table S4). The overall rate of adverse events was not frequent (9.2%), their relevance was relatively low and in none of the patients was meropenem therapy interrupted due to adverse effects. The most frequent events were diarrhea and vomiting (4.2% in the Infusion group vs. 5.8% in the Bolus group and 1.7% vs. 2.5%, respectively); however, the association with meropenem administration was dubious and all events occurred in the period of conversion from parenteral to enteral nutrition. None of the diarrhea events was associated with *Clostridium difficile *infection. The only case of seizures was observed in a patient with traumatic brain injury and ceased immediately after anticonvulsive therapy was established. None of the four episodes of rash were clinically significant and resolved spontaneously. Increased levels of ALT, AST, ALP, bilirubin and thrombocytosis are the most frequently reported laboratory adverse events related to meropenem administration [29,48]. We have observed a significant increase of platelet count in the Bolus group and the rise of ALP in both groups (Additional file 5, Table S5). We found no intergroup difference and no therapeutic measures were necessary. The tolerability profile of meropenem is well established, and the safety of the drug has been extensively reviewed [48,49]; however, to our knowledge, no studies have formally examined and compared adverse effects of continuous or bolus regimens of meropenem.

Our study has several limitations that should be taken into account when interpreting the results. It was not blinded, and was conducted in only one center. The influence of bias in clinical evaluation, despite the third person being involved in case of ambiguity, is indisputable. On the contrary, knowing about this risk of bias, the evaluation of clinical success in the Infusion group could be more strict and censorious. This could explain the discrepancy between insignificant clinical and positive bacteriological results, especially in view of the fact that a microbiologist blinded to the patients' allocation group performed microbiological assessment. Another limitation is frequent concomitant antibiotic therapy (48.1% in the Infusion and 50.9% in the Bolus group) reducing the validity of conclusions about the only impact of meropenem. The dose of meropenem in the Bolus group (6 g/24 hrs) might be potentially confounding, despite that it was used in accordance with the recommendations for the critically ill patients [10,35-39]. Using this dose, the concentration of meropenem could reach T > MIC for 100% of dosing interval in the susceptible pathogens with relatively low MIC. This may potentially favor the Bolus group patients and mask the pharmacodynamic benefit of a continuous regimen. In view of this fact, we recommend for the next studies the use of the same dose of meropenem in both arms adjusted according to the detected MIC. We did not monitor time between the onset of sepsis and administration of antibiotic therapy, which is an important variable correlating with the outcome. Nevertheless, the patients of both groups were treated by the stable team in one ICU, so we could presume that there were no substantial differences between both groups in periods between the onset of sepsis and the start of therapy. We observed shorter meropenem-related ICU LOS in infusion group; however, the ICU discharge criteria were not predefined, which further limits interpretation of our results.

## Conclusions

Continuous infusion of meropenem is safe in comparison with a higher bolus dose, provides equal clinical outcome, generates superior bacteriological efficacy and offers the encouraging alternative of antimicrobial therapy in critically ill patients.

Continuous administration of meropenem was independently associated with favorable microbiological outcome, was connected with shorter length of ICU stay and with fewer days of meropenem therapy.

The reason for failure to demonstrate better clinical cure in the infusion group in spite of the advantageous pharmacodynamic attributes we see in little difference between both types of meropenem administration in achieving T > MIC in relatively susceptible pathogens. The efficacy of continuous or prolonged infusion of meropenem should be investigated in randomized, controlled trials with equal daily doses in both regimens and in populations suffering from infections caused by multi-drug resistant GNB with elevated MICs.

## Key messages

• Continuous infusion of meropenem is safe, in comparison with higher bolus dose, provides equal clinical outcome, generates superior bacteriological efficacy and offers encouraging alternatives of antimicrobial therapy in critically ill patients.

• In this study, continuous administration of meropenem was identified as an independent predictor of microbiological success and was associated with shorter length of ICU stay and with fewer days of meropenem therapy.

## Abbreviations

ALP: alkaline phosphatase; ALT: alanine transaminase; APACHE II: Acute Physiology and Chronic Health Evaluation II; AST: aspartate transaminase; ATB: antibiotic; CFR: cumulative fraction of response; CI: confidence interval; CNS: central nervous system; CRP: C-reactive protein; EOT: end of treatment; GFR: Glomerular filtration rate; GNB: Gram-negative bacteria; ICU: intensive care unit; ITT: intention-to-treat; LOS: length of stay; MRSA: methicillin-resistant staphylococci; MDRD: Modification of Diet in Renal Disease; MIC: Minimum Inhibitory Concentration; n/a: not applicable; OR: odds ratio; PTA: probability of target attainment; RR: Relative risk; ROC: receiver operating characteristic curve; SIRS: systemic inflammatory reactive syndrome; SOFA: Sequential Organ Failure Assessment; VAP: ventilator associated pneumonia; WBC: white blood cell.

## Competing interests

The authors declare that they have no competing interests.

## Authors' contributions

IC was responsible for the study design. MS, PP, AZ, JB, RP and EK were responsible for administering the protocol. IC, MS and JB provided the data analysis and drafted the manuscript. All authors have given final approval of this version of the manuscript.

## Supplementary Material

Additional file 1 **Definitions of different subcategories of outcome**. Detail criteria for evaluation of clinical and microbiological outcome. Click here for file

Additional file 2 **Outcome in ITT and clinically evaluable patients**. Evaluation of secondary outcome measures in ITT patients, in clinically evaluable patients and in subgroups of patients with APACHE II > 20 and MIC ≥ 1.5 mg/l. Click here for file

Additional file 3 **Colonization and superinfection in microbiologically evaluable patients**. The rate of colonization and superinfection and the sorts of identified pathogens in microbiologically evaluable patients. Click here for file

Additional file 4 **Meropenem-related clinical adverse events in ITT population**. The rate and types of adverse events possibly related to meropenem therapy. Click here for file

Additional file 5 **Laboratory parameters related to safety of meropenem therapy in ITT population**. Laboratory parameters at the start and at the end of meropenem therapy. Click here for file

## References

[B1] Devasahayam G Scheld W Hoffman P Newer antibacterial drugs for a new century Expert Opin Investig Drugs 2010 19 215 234 10.1517/13543780903505092 PMC2831224 20053150

[B2] Livermore D Discovery research: the scientific challenge of finding new antibiotics J Antimicrob Chemother 2011 66 1941 1944 10.1093/jac/dkr262 21700626

[B3] Roberts J Lipman J Blot S Rello J Better outcomes through continuous infusion of time-dependent antibiotics to critically ill patients? Curr Opin Crit Care 2008 14 390 396 10.1097/MCC.0b013e3283021b3a 18614901

[B4] Craig WA Basic pharmacodynamics of antibacterials with clinical applications to the use of beta-lactams, glycopeptides, and linezolid Infect Dis Clin North Am 2003 17 479 501 10.1016/S0891-5520(03)00065-5 14711073

[B5] Drusano GL Prevention of resistance: a goal for dose selection for antimicrobial agents Clin Infect Dis 2003 36 S42 S50 10.1086/344653 12516029

[B6] Drusano GL Antimicrobial pharmacodynamics: critical interactions of 'bug and drug' Nat Rev Microbiol 2004 2 289 300 10.1038/nrmicro862 15031728

[B7] Turnidge JD The pharmacodynamics of beta-lactams Clin Infect Dis 1998 27 10 22 10.1086/514622 9675443

[B8] McKinnon PS Paladino JA Schentag JJ Evaluation of area under the inhibitory curve (AUIC) and time above the minimum inhibitory concentration (T > MIC) as predictors of outcome for cefepime and ceftazidime in serious bacterial infections Int J Antimicrob Agents 2008 31 345 351 10.1016/j.ijantimicag.2007.12.009 18313273

[B9] Roberts JA Lipman J Antibacterial dosing in intensive care: pharmacokinetics, degree of disease and pharmacodynamics of sepsis Clin Pharmacokinet 2006 45 755 773 10.2165/00003088-200645080-00001 16884316

[B10] Li C Du X Kuti JL Nicolau DP Clinical pharmacodynamics of meropenem in patients with lower respiratory tract infections Antimicrob Agents Chemother 2007 51 1725 1730 10.1128/AAC.00294-06 17307978 PMC1855547

[B11] Kitzes-Cohen R Farin D Piva G De Myttenaere-Bursztein SA Pharmacokinetics and pharmacodynamics of meropenem in critically ill patients Int J Antimicrob Agents 2002 19 105 110 10.1016/S0924-8579(01)00474-5 11850162

[B12] Novelli A Adembri C Livi P Fallani S Mazzei T De Gaudio AR Pharmacokinetic evaluation of meropenem and imipenem in critically ill patients with sepsis Clin Pharmacokinet 2005 44 539 549 10.2165/00003088-200544050-00007 15871639

[B13] Jaruratanasirikul S Sriwiriyajan S Punyo J Comparison of the pharmacodynamics of meropenem in patients with ventilator-associated pneumonia following administration by 3-hour infusion or bolus injection Antimicrob Agents Chemother 2005 49 1337 1339 10.1128/AAC.49.4.1337-1339.2005 15793108 PMC1068632

[B14] Li C Kuti JL Nightingale CH Nicolau DP Population pharmacokinetic analysis and dosing regimen optimization of meropenem in adult patients J Clin Pharmacol 2006 46 1171 1178 10.1177/0091270006291035 16988206

[B15] Lomaestro BM Drusano GL Pharmacodynamic evaluation of extending the administration time of meropenem using a Monte Carlo simulation Antimicrob Agents Chemother 2005 49 461 463 10.1128/AAC.49.1.461-463.2005 15616337 PMC538854

[B16] Koomanachai P Bulik CC Kuti JL Nicolau DP Pharmacodynamic modeling of intravenous antibiotics against gram-negative bacteria collected in the United States Clin Ther 2010 32 766 779 10.1016/j.clinthera.2010.04.003 20435246

[B17] Lorente L Lorenzo L Martín MM Jiménez A Mora ML Meropenem by continuous versus intermittent infusion in ventilator-associated pneumonia due to gram-negative bacilli Ann Pharmacother 2006 40 219 223 10.1345/aph.1G467 16449546

[B18] Roberts JA Ulldemolins M Lipman J Meropenem: focus on its use in serious bacterial infections Clin Med Rev Ther 2010 2 1 14

[B19] Gonçalves-Pereira J Póvoa P Antibiotics in critically ill patients: a systematic review of the pharmacokinetics of β-lactams Crit Care 2011 15 R206 10.1186/cc10441 21914174 PMC3334750

[B20] Jeurissen A Rutsaert R β-lactam antibiotics in continuous infusion in critically ill patients Crit Care 2010 14 446 10.1186/cc9288 21062510 PMC3219286

[B21] Roberts JA Webb S Paterson D Ho KM Lipman J A systematic review on clinical benefits of continuous administration of beta-lactam antibiotics Crit Care Med 2009 37 2071 2078 10.1097/CCM.0b013e3181a0054d 19384201

[B22] Roberts JA Paratz J Paratz E Krueger WA Lipman J Continuous infusion of beta-lactam antibiotics in severe infections: a review of its role Int J Antimicrob Agents 2007 30 11 18 10.1016/j.ijantimicag.2007.02.002 17442541

[B23] Levy MM Fink MP Marshall JC Abraham E Angus D Cook D Cohen J Opal SM Vincent JL Ramsay G SCCM/ESICM/ACCP/ATS/SIS 2001 SCCM/ESICM/ACCP/ATS/SIS International Sepsis Definitions Conference Crit Care Med 2003 31 1250 1256 10.1097/01.CCM.0000050454.01978.3B 12682500

[B24] Garner JS Jarvis WR Emori TG Horan TC Hughes JM CDC definitions for nosocomial infections, 1988 Am J Infect Control 1988 16 128 140 10.1016/0196-6553(88)90053-3 2841893

[B25] Levey AS Coresh J Balk E Kausz AT Levin A Steffes MW Hogg RJ Perrone RD Lau J Eknoyan G National Kidney Foundation National Kidney Foundation practice guidelines for chronic kidney disease: evaluation, classification, and stratification Ann Intern Med 2003 139 137 147 12859163 10.7326/0003-4819-139-2-200307150-00013

[B26] Nicolau DP Pharmacokinetic and pharmacodynamic properties of meropenem Clin Infect Dis 2008 47 S32 S40 10.1086/590064 18713048

[B27] O'Grady NP Barie PS Bartlett J Practice parameters for evaluating new fever in critically ill adult patients. Task Force of the American College of Critical Care Medicine of the Society of Critical Care Medicine in collaboration with the Infectious Disease Society of America Crit Care Med 1998 26 392 408 10.1097/00003246-199802000-00046 9468180

[B28] Micek ST Ward S Fraser VJ Kollef MH A randomized controlled trial of an antibiotic discontinuation policy for clinically suspected ventilator-associated pneumonia Chest 2004 125 1791 1799 10.1378/chest.125.5.1791 15136392

[B29] Baldwin CM Lyseng-Williamson KA Keam SJ Meropenem: a review of its use in the treatment of serious bacterial infections Drugs 2008 68 803 838 10.2165/00003495-200868060-00006 18416587

[B30] Kuti JL Nightingale CH Knauft RF Nicolau DP Pharmacokinetic properties and stability of continuous-infusion meropenem in adults with cystic fibrosis Clin Ther 2004 26 493 501 10.1016/S0149-2918(04)90051-3 15189746

[B31] Grant EM Kuti JL Nicolau DP Nightingale C Quintiliani R Clinical efficacy and pharmacoeconomics of a continuous-infusion piperacillin-tazobactam program in a large community teaching hospital Pharmacotherapy 2002 22 471 483 10.1592/phco.22.7.471.33665 11939682

[B32] Lorente L Jiménez A Palmero S Jiménez JJ Iribarren JL Santana M Martín MM Mora ML Comparison of clinical cure rates in adults with ventilator-associated pneumonia treated with intravenous ceftazidime administered by continuous or intermittent infusion: a retrospective, nonrandomized, open-label, historical chart review Clin Ther 2007 29 2433 2439 10.1016/j.clinthera.2007.11.003 18158083

[B33] Kojika M Sato N Hakozaki M Suzuki Y Takahasi G Endo S Suzuki K Wakabayasi G A preliminary study of the administration of carbapenem antibiotics in sepsis patients on the basis of the administration time Jpn J Antibiot 2005 58 452 457 10.1038/ja.2005.59 16379157

[B34] Nicasio AM Eagye KJ Nicolau DP Shore E Palter M Pepe J Kuti JL Pharmacodynamic-based clinical pathway for empiric antibiotic choice in patients with ventilator-associated pneumonia J Crit Care 2010 25 69 77 10.1016/j.jcrc.2009.02.014 19427167

[B35] Kuti JL Dandekar PK Nightingale CH Nicolau DP Use of Monte Carlo simulation to design an optimized pharmacodynamic dosing strategy for meropenem J Clin Pharmacol 2003 43 1116 1123 10.1177/0091270003257225 14517194

[B36] Tam VH Schilling AN Neshat S Poole K Melnick DA Coyle EA Optimization of meropenem minimum concentration/MIC ratio to suppress *in vitro *resistance of *Pseudomonas aeruginosa* Antimicrob Agents Chemother 2005 49 4920 4927 10.1128/AAC.49.12.4920-4927.2005 16304153 PMC1315965

[B37] Ludwig E Konkoly-Thege M Kuti JL Nicolau DP Optimising antibiotic dosing regimens based on pharmacodynamic target attainment against *Pseudomonas aeruginosa *collected in Hungarian hospitals Int J Antimicrob Agents 2006 28 433 438 10.1016/j.ijantimicag.2006.07.014 17046212

[B38] Wang H Zhang B Ni Y Kuti JL Chen B Chen M Nicolau DP Pharmacodynamic target attainment of seven antimicrobials against Gram-negative bacteria collected from China in 2003 and 2004 Int J Antimicrob Agents 2007 30 452 457 10.1016/j.ijantimicag.2007.06.005 17646088

[B39] Jaruratanasirikul S Limapichat T Jullangkoon M Aeinlang N Ingviya N Wongpoowarak W Pharmacodynamics of meropenem in critically ill patients with febrile neutropenia and bacteraemia Int J Antimicrob Agents 2011 38 231 236 10.1016/j.ijantimicag.2011.04.019 21726984

[B40] Lodise TP Lomaestro B Drusano GL Piperacillin-tazobactam for *Pseudomonas aeruginosa *infection: clinical implications of an extended-infusion dosing strategy Clin Infect Dis 2007 44 357 363 10.1086/510590 17205441

[B41] Merchant S Gast C Nathwani D Lee M Quintana A Ketter N Friedland I Ingham M Hospital resource utilization with doripenem versus imipenem in the treatment of ventilator-associated pneumonia Clin Ther 2008 30 717 733 10.1016/j.clinthera.2008.04.001 18498921

[B42] Yost R Cappelletty D The Retrospective Cohort of Extended-Infusion Piperacillin-Tazobactam (RECEIPT) Study: a multicenter study Pharmacotherapy 2011 31 767 775 10.1592/phco.31.8.767 21923603

[B43] Roberts JA Kirkpatrick CMJ Roberts MS Robertson TA Dalley AJ Lipman J Meropenem dosing in critically ill patients with sepsis and without renal dysfunction: intermittent bolus versus continuous administration? Monte Carlo dosing simulations and subcutaneous tissue distribution J Antimicrob Chemother 2009 64 142 150 10.1093/jac/dkp139 19398460

[B44] Roberts JA Boots R Rickard CM Thomas P Quinn J Roberts DM Richards B Lipman J Is continuous infusion ceftriaxone better than once-a-day dosing in intensive care? A randomized controlled pilot study J Antimicrob Chemother 2007 59 285 291 17135183 10.1093/jac/dkl478

[B45] Lubasch A Lück S Lode H Mauch H Lorenz J Bölcskei P Welte T COPD Study Group Optimizing ceftazidime pharmacodynamics in patients with acute exacerbation of severe chronic bronchitis J Antimicrob Chemother 2003 51 659 664 10.1093/jac/dkg111 12615868

[B46] Nicolau DP McNabb J Lacy MK Quintiliani R Nightingale CH Continuous versus intermittent administration of ceftazidime in intensive care unit patients with nosocomial pneumonia Int J Antimicrob Agents 2001 17 497 504 10.1016/S0924-8579(01)00329-6 11397621

[B47] Lau WK Mercer D Itani KM Nicolau DP Kuti JL Mansfield D Dana A Randomized, open-label, comparative study of piperacillin-tazobactam administered by continuous infusion versus intermittent infusion for treatment of hospitalized patients with complicated intra-abdominal infection Antimicrob Agents Chemother 2006 50 3556 3561 10.1128/AAC.00329-06 16940077 PMC1635208

[B48] Linden P Safety profile of meropenem: an updated review of over 6,000 patients treated with meropenem Drug Saf 2007 30 657 668 10.2165/00002018-200730080-00002 17696578

[B49] Norrby SR Gildon KM Safety profile of meropenem: a review of nearly 5,000 patients treated with meropenem Scand J Infect Dis 1999 31 3 10 10.1080/00365549950161808 10381210

