# Supplementary material for: Clinical and microbiological efficacy of continuous versus intermittent application of meropenem in critically ill patients: a randomized open-label controlled trial
Source: Crit Care. 2012 Jun 28;16(3):R113. doi: 10.1186/cc11405 (PMC3580671; doi:10.1186/cc11405)
Supplement: Additional file 1 — Definitions of different subcategories of outcome. Detail criteria for evaluation of clinical and microbiological outcome. [file cc11405-S1.DOC]

Additional file 1

Title: Definitions of different subcategories of outcome

Description: Detail criteria for evaluation of clinical and microbiological outcome

| Clinical evaluation | Clinical Success | Cure | A complete resolution of all acute signs and symptoms of infection, with no new signs or symptoms associated with the original infection |
| --- | --- | --- | --- |
|  |  | Improved | Patient who retained evidence of infection but demonstrated a reduction of the majority of the clinical signs and symptoms of infection and no new or worsened signs associated with the original infection |
|  |  |  | Patient improved during therapy but died from a noninfectious process |
|  | Clinical Failure | Failure | A persistence or progression of signs and symptoms of infection, development of new clinical findings consistent with active infection, or death from infection |
| Microbiological evaluation | Microbiological success | Eradication | An elimination of the pathogen from the site of isolation |
|  |  | Presumed eradication | An absence of appropriate material for culture or absence of results of control microbiological tests coupled with clinical improvement after a pathogen was initially isolated |
|  |  | Colonization | Detection of new pathogen from the site of infection during therapy without need for antimicrobial treatment or a superinfection with a microbiological agent outside the treatment spectrum of meropenem (G+/fungi) |
|  | Microbiological Failure | Verified persistence | The failure to eradicate the original pathogen from the site of isolation after completion of therapy |
|  |  | Presumed persistence | absence of appropriate material for culture or absence of results of control microbiological tests coupled with lack of clinical improvement after a pathogen was initially isolated |
|  |  | Resistance | Development of resistance to meropenem during therapy |
|  |  | Superinfection | Detection of a new pathogen from the site of infection during therapy with need for antimicrobial treatment |
|  | Microbiological unevaluable | Unevaluable | Patients without cultures or evident pathogens from the presumed site of infection |
