# Supplementary material for: Clinical and microbiological efficacy of continuous versus intermittent application of meropenem in critically ill patients: a randomized open-label controlled trial
Source: Crit Care. 2012 Jun 28;16(3):R113. doi: 10.1186/cc11405 (PMC3580671; doi:10.1186/cc11405)
Supplement: Additional file 2 — Outcome in ITT and clinically evaluable patients. Evaluation of secondary outcome measures in ITT patients, in clinically evaluable patients and in subgroups of patients with APACHE II > 20 and MIC ≥ 1.5 mg/l. [file cc11405-S2.DOC]

Additional file 2

Title: Outcome in ITT and clinically evaluable patients

Description: Evaluation of secondary outcome measures in ITT patients, in clinically evaluable patients and in subgroups of patients with APACHE II >20 and MIC ≥1.5 mg/l.

| **Parameter** | **Infusion group** | **Bolus group** | ***P* value** |
| --- | --- | --- | --- |
| Number of patients |  |  | 0,695 |
| ITT | 120 | 120 |  |
| Clinically evaluable | 106 | 108 |  |
| APACHE II >20 | 49 | 53 | 0.684 |
| MIC ≥1.5 mg/l | 14 | 21 | 0.268 |
| Meropenem-related length of mechanical ventilation |  |  |  |
| ITT | 9 (5-13) | 11 (6-17) | 0.051 |
| Clinically evaluable | 9 (5–15) | 12 (6–19) | 0.058 |
| APACHE II >20 | 9 (7-12) | 11 (6-16) | 0.434 |
| MIC ≥1.5 mg/l | 9 (6-13) | 12 (5-17.5) | 0,614 |
| Meropenem-related ICU LOS |  |  |  |
| ITT | 10 (7-14) | 12 (7-19) | **0.044** |
| Clinically evaluable | 10 (7–16.5) | 13 (8–21) | **0.042** |
| APACHE II >20 | 11 (9-12.5) | 11.5 (7-18) | 0,529 |
| MIC ≥1.5 mg/l | 10.5 (8.5-15) | 13 (5-19 | 0,894 |
| Meropenem-related hospital LOS |  |  |  |
| ITT | 26 (17-38) | 22 (12-35) | 0.079 |
| Clinically evaluable | 28 (18–39) | 25 (14–42) | 0.412 |
| APACHE >20 | 25.5 (18-38.5) | 21.5 (13-36) | 0,314 |
| MIC ≥1.5 mg/l | 28 (22-42) | 26 (15-33) | 0.657 |
| ICU mortality |  |  |  |
| ITT | 18 (15.0%) | 25 (20.8%) | 0.313 |
| Clinically evaluable | 14 (11.6%) | 17 (14.2%) | 0.701 |
| APACHE II >20 | 9 (20.5%) | 6 (14.3%) | 0.573 |
| MIC ≥1.5 mg/l | 4 (19%) | 1 (7.1%) | 0,627 |
| Hospital mortality |  |  |  |
| ITT | 21 (17.5%) | 28 (23,3%) | 0.337 |
| Clinically evaluable | 17 (16.0%) | 19 (15.7%) | 0.857 |
| APACHE II >20 | 13 (29.5%) | 10 (23.8% | 0,630 |
| MIC ≥1.5 mg/l | 4 (19%) | 1 (7.1%) | 0,627 |
| Leucocytes^a^ (x 10^9^) at EOT |  |  |  |
| ITT | - | - | n/a |
| Clinically evaluable | 10.7 (8.7-12.1) | 11.2 (8.6-16.2) | 0.340 |
| APACHE II >20 | 11.5 (9.5-14.4) | 12.3 (9.2-16.2) | 0.607 |
| MIC ≥1.5 mg/l | 12.5 (8.5-13.5) | 15.3 (10.7-18.2) | 0.174 |
| CRP^a^ (mg/l) at EOT |  |  |  |
| ITT | - | - | n/a |
| Clinically evaluable | 59 (32-87) | 72 (37-114) | **0.037** |
| APACHE II >20 | 62 (32-87) | 78 (42-126) | **0,036** |
| MIC ≥1.5 mg/l | 43.5 (39-93) | 100 (37-134) | 0.279 |

Values are presented as absolute (percentage) or median (interquartile range). ITT, intention-to-treat; APACHE II, Acute Physiology and Chronic Health Evaluation II; MIC, minimum inhibitory concentration; ICU, intensive care unit; LOS, length of stay; EOT, end of treatment; n/a, not applicable; ^a^ post hoc analysis; CRP, C-reactive protein
