# Supplementary material for: Clinical and microbiological efficacy of continuous versus intermittent application of meropenem in critically ill patients: a randomized open-label controlled trial
Source: Crit Care. 2012 Jun 28;16(3):R113. doi: 10.1186/cc11405 (PMC3580671; doi:10.1186/cc11405)
Supplement: Additional file 3 — Colonization and superinfection in microbiologically evaluable patients. The rate of colonization and superinfection and the sorts of identified pathogens in microbiologically evaluable patients. [file cc11405-S3.DOC]

Additional file 3

Title: Colonization and superinfection in microbiologically evaluable patients

Description: The rate of colonization and superinfection and the sorts of identified pathogens in microbiologically evaluable patients.

|  | **Infusion (n = 96)**  **n (%)** | **Bolus (n =102)**  **n (%)** | ***P* value** |
| --- | --- | --- | --- |
| **Colonization overall** | **14 (14.6%)** | **19 (18.6%)** | 0.568 |
| *Acinetobacter spp.* | 1 (1.0%) | 3 (2.9%) | 0.622 |
| *Burkholderia cepacia* | 2 (2.1%) | 0 | 0.239 |
| *Pseudomonas aeruginosa* | 2 (2.1%) | 3 (2.9%) | 1.000 |
| *Stenotrophomonas maltophilia* | 1 (1.0%) | 3 (2.9%) | 0.622 |
| *Ralstonia picketti* | 1 (1.0%) | 2 (2.0%) | 1.000 |
| **Superinfection overall** | **7 (7.3%)** | **8 (7.8%)** | 1.000 |
| *Candida albicans* | 6 (6.3%) | 8 (7.8%) | 0.784 |
| *Aspergillus species* | 1 (1.0%) | 0 | 0.485 |

Values are presented as absolute (percentage).
