# Supplementary material for: Clinical and microbiological efficacy of continuous versus intermittent application of meropenem in critically ill patients: a randomized open-label controlled trial
Source: Crit Care. 2012 Jun 28;16(3):R113. doi: 10.1186/cc11405 (PMC3580671; doi:10.1186/cc11405)
Supplement: Additional file 4 — Meropenem-related clinical adverse events in ITT population. The rate and types of adverse events possibly related to meropenem therapy. [file cc11405-S4.DOC]

Additional file 4

Title: Meropenem-related clinical adverse events in ITT population

Description: The rate and types of adverse events possibly related to meropenem therapy

|  | **Infusion (n =120)**  **n (%)** | **Bolus (n =120)**  **n (%)** | ***P* value** |
| --- | --- | --- | --- |
| Diarrhea | 5 (4.2%) | 7 (5.8%) | 0.769 |
| Rash | 3 (2.5%) | 1 (0.8%) | 0.622 |
| Vomiting | 2 (1.7%) | 3 (2.5%) | 1.000 |
| Seizures | 0 (0.0%) | 1 (0.8%) | 1.000 |

Values are presented as absolute (percentage) or mean ± standard deviation or median (interquartile range). ITT, intention-to-treat.
