# Supplementary material for: Clinical and microbiological efficacy of continuous versus intermittent application of meropenem in critically ill patients: a randomized open-label controlled trial
Source: Crit Care. 2012 Jun 28;16(3):R113. doi: 10.1186/cc11405 (PMC3580671; doi:10.1186/cc11405)
Supplement: Additional file 5 — Laboratory parameters related to safety of meropenem therapy in ITT population. Laboratory parameters at the start and at the end of meropenem therapy. [file cc11405-S5.DOC]

Additional file 5

Title: Laboratory parameters related to safety of meropenem therapy in ITT population

Description: Laboratory parameters at the start and at the end of meropenem therapy

|  | **Start of meropenem therapy** | |  | **End of meropenem therapy** | |  |
| --- | --- | --- | --- | --- | --- | --- |
|  | Infusion  (n =120) | Bolus  (n =120) | p | Infusion  (n =120) | Bolus  (n =120) | p |
| Bilirubin (µmol/l) | 16.0 (9.0-34.0) | 19.5 (12.0-33.0) | 0.420 | 12 (9.0-21.0) | 16.0 (9.0-27.0) | 0.070 |
| ALT (µkat/l) | 0.83 (0.50-1.78) | 0.97 (0.47-1.72) | 0.865 | 0.93 (0.51-1.78) | 1.24 (0.71-2.03) | 0.051 |
| AST (µkat/l) | 0.89 (0.47-1.31) | 0.96 (0.54-1.56) | 0.478 | 0.65 (0.49-1.23) | 0.84 (0.55-1.29) | 0.066 |
| ALP (µkat/l) | 2.12 (1.35-3.22) | 2.09 (1.31-3.59) | 0.701 | 2.71 (1.84-4.54)^a^ | 3.29 (2.02-4.46)^a^ | 0.627 |
| Thrombocytes (x 10^9^/l) | 277 (183-422) | 239 (151-393) | 0.147 | 312 (270-327) | 352 (230-403)^a^ | 0.088 |

Values are presented as absolute (percentage) or mean ± standard deviation or median (interquartile range). ^a^ p<0,001, - comparison between the start and the end of therapy (Wilcoxon signed-rank test). ITT, intention-to-treat; ALT, [alanine transaminase](http://en.wikipedia.org/wiki/Alanine_transaminase); AST, aspartate transaminase; ALP, alkaline phosphatase.
